# Comprehensive Data-Driven Assessment of Non-Kinase Targets of Inhibitors of the Human Kinome

**DOI:** 10.3390/biom14030258

**Published:** 2024-02-21

**Authors:** Mona Mobasher, Martin Vogt, Elena Xerxa, Jürgen Bajorath

**Affiliations:** LIMES Program Unit Chemical Biology and Medicinal Chemistry, Department of Life Science Informatics and Data Science, B-IT, Lamarr Institute for Machine Learning and Artificial Intelligence, University of Bonn, Friedrich-Hirzebruch-Allee 5/6, 53115 Bonn, Germany

**Keywords:** protein kinases, human kinome, kinase inhibitors, activity data, multi-target activity, non-kinase targets

## Abstract

Protein kinases (PKs) are involved in many intracellular signal transduction pathways through phosphorylation cascades and have become intensely investigated pharmaceutical targets over the past two decades. Inhibition of PKs using small-molecular inhibitors is a premier strategy for the treatment of diseases in different therapeutic areas that are caused by uncontrolled PK-mediated phosphorylation and aberrant signaling. Most PK inhibitors (PKIs) are directed against the ATP cofactor binding site that is largely conserved across the human kinome comprising 518 wild-type PKs (and many mutant forms). Hence, these PKIs often have varying degrees of multi-PK activity (promiscuity) that is also influenced by factors such as single-site mutations in the cofactor binding region, compound binding kinetics, and residence times. The promiscuity of PKIs is often—but not always—critically important for therapeutic efficacy through polypharmacology. Various in vitro and in vivo studies have also indicated that PKIs have the potential of interacting with additional targets other than PKs, and different secondary cellular targets of individual PKIs have been identified on a case-by-case basis. Given the strong interest in PKs as drug targets, a wealth of PKIs from medicinal chemistry and their activity data from many assays and biological screens have become publicly available over the years. On the basis of these data, for the first time, we conducted a systematic search for non-PK targets of PKIs across the human kinome. Starting from a pool of more than 155,000 curated human PKIs, our large-scale analysis confirmed secondary targets from diverse protein classes for 447 PKIs on the basis of high-confidence activity data. These PKIs were active against 390 human PKs, covering all kinase groups of the kinome and 210 non-PK targets, which included other popular pharmaceutical targets as well as currently unclassified proteins. The target distribution and promiscuity of the 447 PKIs were determined, and different interaction profiles with PK and non-PK targets were identified. As a part of our study, the collection of PKIs with activity against non-PK targets and the associated information are made freely available.

## 1. Introduction

In pharmaceutical research, protein kinases (PKs) are among the most intensely investigated drug targets [1,2]. PKs catalyze the adenosine triphosphate (ATP)-cofactor-dependent phosphorylation of tyrosine, serine, or threonine residues in proteins (including PKs) that participate in intracellular signal transduction [1]. Tyrosine PKs and serine/threonine PKs represent the two major PK groups. The active and cofactor binding sites of PKs are located in their catalytic domain, whose activity is controlled by interactions with the associated regulatory domain. PK-mediated signaling generally depends on the formation of phosphorylation cascades involving PKs and other proteins. These signaling pathways often originate from membrane receptor-associated PKs that transduce extracellular signals into cells. Aberrant or deregulated signaling events are responsible for many different diseases. Uncontrolled PK activity can be treated with small-molecular PK inhibitors (PKIs), synthetic compounds that have become prime drug candidates [1,2]. The majority of PKIs bind non-covalently to the ATP cofactor binding site that is largely conserved across human PKs or proximal to this site [1,3]. If changes in the ATP binding site occur, they are typically limited to replacements of one or a few residues. ATP-site-directed PKIs are either ATP-competitive, representing a “classical” type of PKIs, or induce local structural changes by binding adjacent to the ATP site, which stabilizes an inactive conformational state of PKs [1,3]. 

The first human (tyrosine) PKs were discovered in 1980 [4], followed by intense searches for other PKs. Only 15 years later, the PK superfamily was introduced [5]. Then, in 2002, in the course of the human genome project, 518 PKs comprising the kinome were identified [6]. Accompanying advances in PK biology, in the mid-1980s, the first small-molecular PKIs were identified and used to investigate PK functions, thereby establishing PKs as pharmaceutical targets [7]. These investigations were complemented by extensive structural studies of PKs and their complexes with PKIs, yielding a wealth of X-ray structures [8] that uncovered different binding modes of PKIs [3] and substantially aided in their optimization [9]. 

PKIs were considered as candidate compounds in different therapeutic areas such as cancer [10,11], immunology and inflammation [12], diseases of the central nervous system [13], or metabolic diseases [14]. In 2001, the first PKI (imatinib) was approved as a drug in oncology for the treatment of leukemia [15,16], which further intensified and accelerated PK drug discovery, especially in oncology [16]. Currently, 80 PKIs are marketed as drugs for cancer treatment and other therapeutic applications [17,18]. The high interest in PKIs as drug candidates has also led to a wealth of compound data. For instance, more than 150,000 PKIs with high-confidence activity data and target annotations have become publicly available covering more than 80% of the human kinome [19,20]. Hence, these compounds and their associated activity data provide a substantial knowledge base for the characterization of PKIs and their target distribution. Although increasing numbers of allosteric PKIs are reported that bind to different sites in the catalytic PK domain and elicit conformational effects by non-covalent or covalent mechanisms, they are still very rare compared to ATP-site-directed PKIs [21].

When PKIs were first developed as drug candidates during the late 1980s and 1990s, molecular biology-driven reductionist approaches governed drug discovery [22]. According to the reductionist approach, biological systems were decomposed into their molecular components, the individual targets implicated in diseases were cloned and expressed, and target-based assays were developed to search for compounds that specifically inhibited or modulated a given target [22]. Given the strong focus on individual targets, many candidate compounds identified using reductionist approaches were originally thought to be target-specific or -selective, which also applied to imatinib and other PKIs first developed for cancer therapy [16]. During the early days of PK drug discovery, when kinome screens and chemoproteomic assays were not yet available, new candidate inhibitors were only tested in a few assays for other PKs. However, in many instances, it was subsequently discovered that PKIs inhibited multiple PKs and that their clinical efficacy in cancer treatment depended on simultaneous interference with multiple signaling pathways [11,16]. 

Corresponding findings were also reported for other targets and active compounds. Beginning in the late 1990s, increasing evidence was accumulating, through high-throughput screening and target profiling campaigns, that subsets of drugs interacted with multiple targets and that multi-target (MT) engagement was often decisive for their therapeutic efficacy. At the same time, the presence of overlapping or interdependent pharmacological networks was increasingly recognized [23] as pharmacology continued to evolve into a molecular science. Taken together, the notion of frequent MT activity of drugs on the one hand and network pharmacology on the other gave rise to the concept of “polypharmacology”, formally introduced in 2006 [24], referring to the use of compounds with MT activity for the treatment of complex multi-factorial diseases and the ensuing pharmacological effects [24,25,26]. Importantly, these MT activities resulted from “multi-specific” ligand–target interactions that must be clearly distinguished from non-specific effects.

The first generation of PKIs developed for cancer treatment became a paradigm for polypharmacology in drug discovery [11]. Polypharmacology further evolved over the next decade and became an established drug discovery strategy [24,25,26,27,28], complementing or even replacing the development of target-specific compounds in different therapeutic areas. However, polypharmacology was not always desirable and had a downside because the MT activity of drugs was also responsible for adverse (side) effects by engaging targets other than those intended [25,26]. Adverse-effect risk assessment represents a critical issue in drug development. In the case of life-threatening diseases, adverse effects might be tolerated. In other situations, such as long-term treatment of chronic diseases, adverse effects must inevitably be minimized, calling for target-specific drug candidates. 

Since most PKIs are directed against the largely conserved ATP site, multi-kinase activity of PKIs might be anticipated. Therefore, the view of PKI activity changed after the polypharmacological nature of first-generation clinical PKIs was discovered. Accordingly, PKIs were generally expected to be active against several PKs. However, this view was subsequently not supported by large-scale kinome profiling assays of PKIs and chemoproteomic studies [29,30,31], which confirmed the presence of many target-specific or -selective PKIs in addition to promiscuous inhibitors. For example, Klaeger et al. determined the cellular targets of 243 clinical PKIs, revealing a variety of activity profiles ranging from kinase-specific to highly promiscuous compounds [31]. Hence, the multi-kinase activity of PKIs cannot be generally assumed, and subtle changes in ATP binding, varying PK plasticity and dynamics, and/or differences in compound binding kinetics likely cause distinct selectivity vs. promiscuity profiles of many PKIs [1,32]. 

While it is not unusual that active compounds bind to closely related targets, MT activity might also involve distantly related or unrelated targets. Although activity against secondary targets is often responsible for adverse effects, it also provides the basis for drug repurposing, that is, finding new therapeutic applications for approved drugs [33]. Activity of PKIs against non-PK targets has also been observed on a case-by-case basis [31,34]. For example, PKIs have been shown to bind to bromodomains [35], and dual PK and bromodomain inhibitors have been generated for polypharmacology [36]. Bromodomain-containing proteins bind to acetylated lysine residues in chromatic histone tails and are involved in the regulation of gene transcription. In addition, PKIs also inhibit functionally distinct metabolic enzymes such as indolamin-2,3-dioxygenase or NRH:quinone oxidoreductase 2 [34]. Furthermore, while G-protein-coupled receptor (GPCR) associated PKs selectively phosphorylate the intracellular domain of GPCRs during signal transduction [37], PKIs were also found to bind to aminergic GPCRs [38,39]. 

Given the wealth of PKIs and activity data that have become publicly available, we carried out a systematic analysis of non-PK targets of PKIs on the basis of curated activity data, as reported in the following. The data-driven analysis provides a comprehensive account of secondary PKI targets implicated in polypharmacology and identifies PKIs with activity against different combinations of PKs and other non-PK targets. 

## 2. Materials and Methods

### 2.1. Targets, Compounds, and Activity Data

A set of 155,579 human PKIs was obtained from a recently conducted large-scale analysis of human and mouse PKIs from public sources [21]. These human PKIs were active against a total of 440 PKs, representing ~85% of the human kinome. For these PKIs, activity data were curated from ChEMBL [40] (version 31) and BindingDB [41] (accessed on 20 October 2022). PKs were aggregated on the basis of their UniProt IDs [42]. Only PKIs with available IC_50_, K_i_, and/or K_d_ potency measurements and standard activity relationship “=” were considered. Potency values were recorded in negative decadic logarithmic form. Preliminary measurements such as “% inhibition” that typically result from single-point (concentration) screening experiments were not considered to ensure reliability of the target annotations. In addition, (assay-dependent) IC_50_ and (assay-independent) K_i_ or K_d_ values (equilibrium constants) cannot not be directly compared and were separately considered. This is particularly relevant for IC_50_ values from PKI assays conducted with varying ATP concentrations that do not correspond to physiological conditions. 

As a threshold for minimal PKI activity, a value of 10 µM was applied (corresponding to a negative decadic logarithmic (pPot) value of 5). Notably, for PKIs, activity at 10 µM concentration against PKs is considered rather weak from a medicinal chemistry viewpoint. However, this threshold was applied, exclusively based on high-confidence measurements, to ensure consistency with non-PK targets, where micromolar activities should be considered in MT activity analysis. The consideration of weak PK activities has additional implications, as further discussed below. 

PKIs were aggregated based on non-stereo-sensitive canonical SMILES [43] representations after standardization, including neutralization, elimination of salts, and removal of stereochemical information. If multiple potency annotations of the same type were available for a PKI, they were averaged if all values fell into the same order of magnitude, while PKIs with inconsistent or contradictory annotations were removed [21]. 

Analogously to PKs, to identify human non-PK targets of PKIs, the SMILES representations of the curated PKI data set were used to systematically query ChEMBL and BindingDB for additional non-PK activity data. For consistency, the same versions of ChEMBL and BindingDB as above were used. For ChEMBL, target identifiers were required to belong to the SINGLE PROTEIN target type and “Homo sapiens” organism. Only records from direct binding assays (relationship type D) at the highest level of confidence (score 9) were considered in the presence of standard activity relationship “=” and IC_50_, K_i_, and/or K_d_ potency measurement reported in nM (standard unit “nM”). Additionally, PKIs containing multiple annotations against the same target with inconclusive or contradictory annotations such as “inactive” or “inconclusive” were discarded. Corresponding criteria were applied to query BindingDB; that is, only human single-protein chain targets were considered, and IC_50_, K_i_, and/or K_d_ with standard relation “=” were required as potency measurements. For non-PK targets from both databases, a minimal potency of 10 µM was required to ensure consistency with the analysis of PK activities, as stated above.

To eliminate possible false-positive activity annotations, potential pan assay interference compounds [44] and compounds violating Eli Lilly Medicinal Chemistry rules for chemical integrity [45] were removed. In addition, compounds with reported activity against anti-targets or other undesired targets such as hERG, cytochrome P450 isoforms, or albumin were omitted. 

Non-PK activity annotations of PKIs were also converted into negative decadic logarithmic values (pIC_50,_ pK_i_, and pK_d_), which were subsequently averaged if replicates of the same measurement type were reported. Averaged values with standard deviation larger than 1 were discarded. For data aggregation, interactions with assay-independent pK_i_ and pK_d_ measurement were prioritized over pIC_50_ values, and the highest reported value was used as the final activity annotation.

For PKIs, a PK promiscuity degree (PK_PD) was calculated as the number of PKs against which a given PKI was active. Analogously, a non-PK promiscuity degree (Non-PK_PD) was calculated as the number of non-PK targets a of PKI. 

### 2.2. Protein Classification

Non-PK targets were classified using ChEMBL protein classification levels 1 and 2. Level 1 comprises the classes enzyme, membrane receptor, epigenetic regulator, ion channel, transcription factor, transporter, secreted protein, cytosolic other protein, “other”, and “unclassified”. Enzymes and membrane receptors were further divided according to level 2 into different target types (for example, reductase, protease, hydrolase, or transferase enzymes). 

### 2.3. Kinome Tree Mapping and PKI-Based Target Network

Human PKs sharing inhibitors with non-PK targets were mapped onto a phylogenetic tree representation of the human kinome using KinMap [46]. 

To assess PKI-based target relationships, a PKI-based target network was generated, in which PKs and non-PK targets were represented as nodes that were connected by an edge if two targets shared at least two PKIs. Nodes were scaled in size according to their degree, that is, the number of edges (relationships with other targets).

## 3. Results and Discussion

### 3.1. Data Curation 

As detailed above, systematic analysis of PKI targets was based on a recently generated collection of ~155,000 human PKIs for which high-confidence activity data were available, and additional confidence criteria were applied. Stringent activity data analysis was carried out to ensure that the analysis of PKI targets was exclusively based on most reliable measurements in order to avoid false-positive target annotations. The application of stringent data confidence criteria might also lead to some false negatives (that is, true targets for which only preliminary assay results were available). However, such incidences are generally less likely than false positives and can be tolerated. By contrast, false positives represent the major source of errors in promiscuity assessment and should strictly be avoided. 

### 3.2. Protein Kinase Inhibitors with Non-Kinase Targets 

On the basis of our large-scale analysis, we identified a total 447 PKIs with activity against non-PK targets. These 447 PKIs were active with at least 10 µM potency against a total of 390 human PKs and 210 non-PK targets. Hence, for only ~0.3% of PKIs with available high-confidence activity data, additional non-PK targets were identified, reflecting the need for a comprehensive data-driven assessment. However, the total number of PKIs with non-PKI targets uncovered by our data analysis was larger than we initially anticipated, providing a sound basis for further analysis. 

### 3.3. Promiscuity Assessment

**Figure 1:**
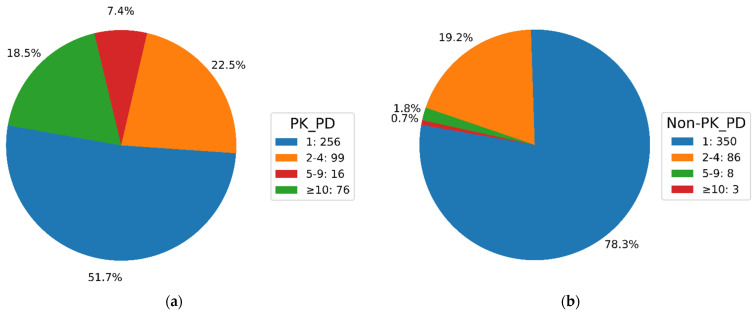
Promiscuity degrees. For PKIs with non-PK targets, pie charts show the distributions of PDs for (**a**) human PKs and (**b**) non-kinase targets.

**Figure 2:**
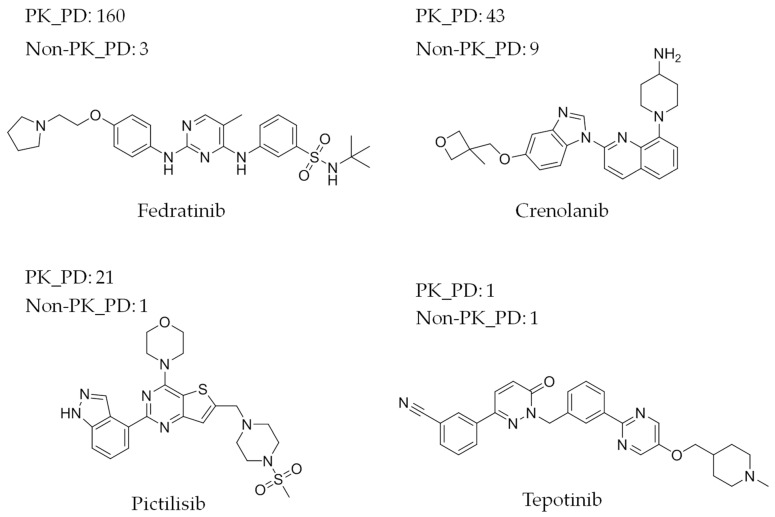
Exemplary protein kinase inhibitors with activity against other targets. Shown are clinical PKIs with varying degrees of PK and non-PK target promiscuity.

For the 447 PKIs, the numbers of PK and non-PK targets (PK_PD and Non-PK_PD values) were determined. Figure 1a shows the results for PK targets. More than half of these PKIs (256, corresponding to 57.3%) were reported to be active against a single PK, while 99 (22.1%) were active against two to four PKs. Moreover, 76 PKIs (17%) were annotated with 10 or more PKs. These PK numbers were on average higher than observed for the original collection of ~155,000 human PKIs [20]. Hence, while the very different sample sizes of the original set and the PKIs with non-PK targets precluded a more detailed statistical comparison, there was a notable enrichment of PKIs with above-average PK promiscuity among the 447 PKIs. Figure 1b shows the corresponding results for non-PK targets. Here, a partly different picture emerged. The majority of PKIs (350, 78.3%) were only reported to be active against a single non-PK target, while 86 (19.2%) were annotated with two to four non-PK targets. However, only 11 of the 447 PKIs were active against larger numbers of targets. Thus, PKIs with single non-PK targets clearly dominated the distribution. Figure 2 shows exemplary PKIs with varying promiscuity against PKs and other targets. 

### 3.4. Distribution of Non-Protein Kinase Targets

**Figure 3:**
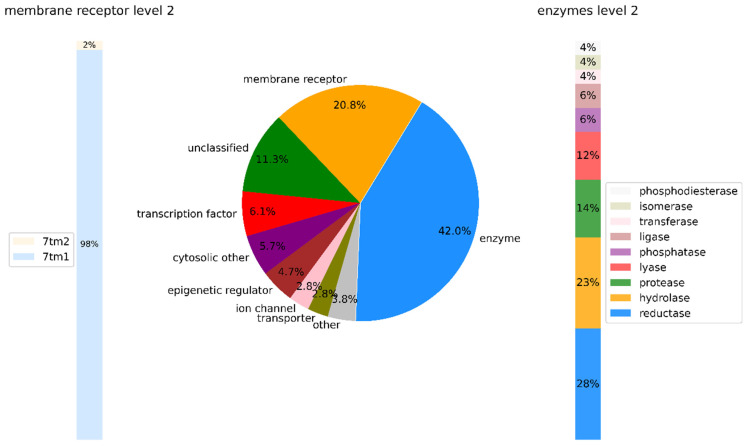
Classification of non-protein kinase targets. Shown are the level 1 (target class) assignments according to the ChEMBL target classification scheme for non-PK targets of the 447 PKIs. Enzyme and membrane receptor targets were further divided into different target types (level 2).

Non-PK targets were grouped into different target classes. Figure 3 shows the distribution of PKI targets across these classes. Non-PK targets belonging to nine ChEMBL classes were detected. Thus, the 447 PKIs were active against a wide variety of non-PKI targets. With 42% of all targets, enzymes were prevalent among non-PK targets, followed by membrane receptors (20.8%) and unclassified proteins (often originating from human genome analysis and being little characterized functionally thus far). 

Enzyme targets belonged to nine different types. Reductases (28%) and hydrolases (23%) were most frequently observed, followed by proteases (14%). By contrast, nearly all (98%) of the membrane receptor targets belonged to the seven transmembrane type 1 (7tm1) categories, which comprised a variety of (class A) GPCR families. Among others, these included popular pharmaceutical targets such as serotonin, acetylcholine, opioid, or adrenergic receptors. 

### 3.5. Inhibitor–Target Interactions

**Figure 4:**
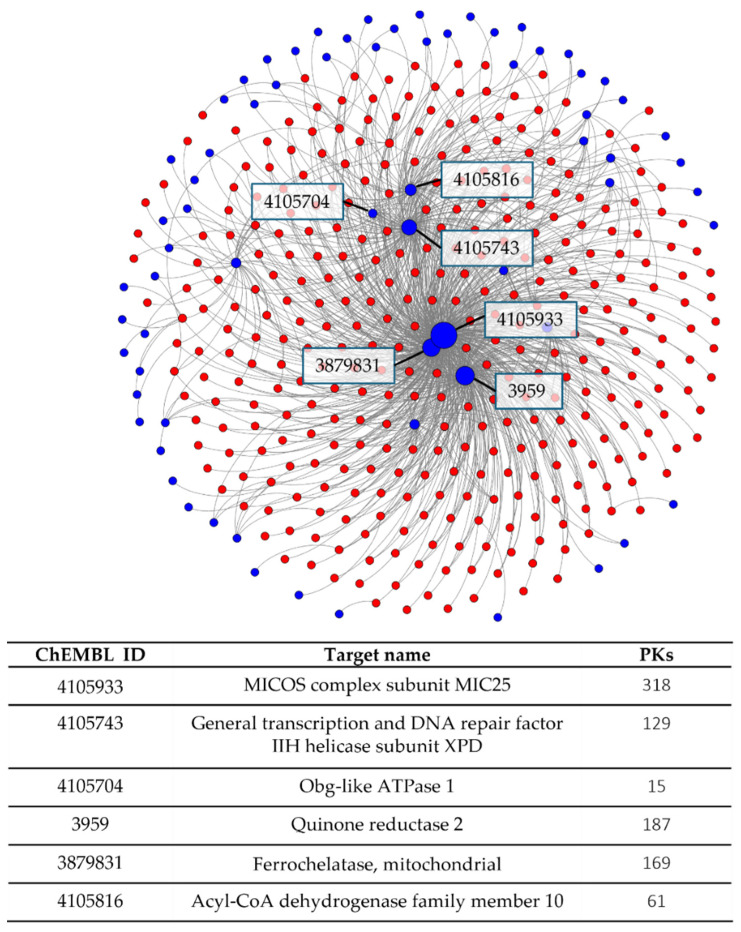
Protein-kinase-inhibitor-based target network. Red nodes represent PKs and blue nodes non-PK targets. Edges were drawn between targets if they shared at least two PKIs. Nodes were scaled in size according to the number of edges they formed. Exemplary non-PK targets were labeled. Target names and the number of connected PKs are reported in the table insert at the bottom.

A PKI-based target network was generated to globally analyze and visualize the distribution of PKI interactions with PK and non-PK targets. In the network shown in Figure 4, targets were connected if they shared at least two PKIs. This network contained 342 unique PKs and 69 unique non-PK targets connected by a total of 1120 edges. The network representation highlighted the prevalence of PKIs with activity against single-PK and non-PKI targets and also revealed the presence of several non-PKI targets sharing PKIs with many different PKs, resulting in large blue nodes in the center of the network. 

### 3.6. Kinase Targets

**Figure 5:**
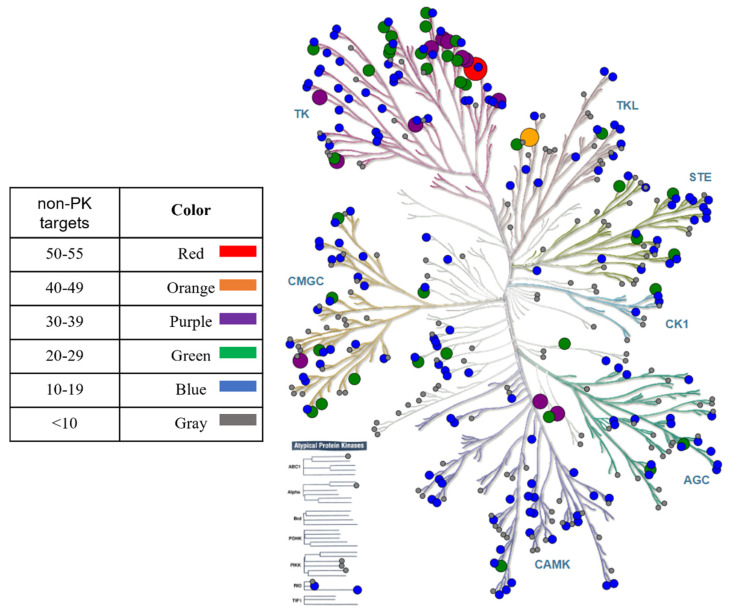
Phylogenetic tree mapping of protein kinases sharing inhibitors with non-kinase targets. PKs are represented as nodes that are scaled in size and color coded according to the number of non-PK targets of shared PKIs.

Next, the distribution of PK targets of PKIs shared with non-PK targets was determined. In Figure 5, the 390 PK targets of the 447 PKIs were mapped on a phylogenetic tree representation of the human kinome. The PK targets were widely distributed across the kinome involving all PK groups. PKs sharing PKIs with largest numbers of non-PK targets preferentially belonged to the tyrosine PK (TK) group. In addition, individual PKs sharing large numbers of PKIs with non-PK targets were also found in the tyrosine PK-like (TKL) group as well as the CMGC and CAMK groups of serine/threonine PKs. 

The TK group is the most intensely studied PK group, followed by the CMGC/CAMK groups. This might explain why most PK targets of PKIs shared with non-PK targets wereidentified for these groups, as also reported in Table 1. 

In addition, Table 1 reports PKs with largest numbers of PKIs shared with non-PK targets. For the vascular endothelial growth factor receptor 2 and epidermal growth factor receptor erbB1 PKs (both belonging to the TK group), more than 50 shared PKIs were identified. Furthermore, a total of 13 PKs and 38 PKs from different groups shared at least 30 and 20 PKIs with non-PK targets, respectively. Moreover, 162 PKs shared 10 or more PKIs with other targets. Thus, while the majority of shared PKIs was annotated with singlePK and non-PK targets, as discussed above, a large number of PKs shared multiple PKIs with different non-PK targets, hence revealing a variety of PKI target combinations. 

### 3.7. Most Frequent Non-Protein Kinase Targets

In light of these findings, the non-PK targets with largest numbers of active PKIs were determined and found to include a variety of functionally unrelated targets. Table 2 reports the top 10 non-PK targets that shared 15–32 inhibitors with different PKs. The largest number of PKIs was identified for acetylcholinesterase (32 PKIs), followed by an as of yet uncharacterized protein (FLJ45252, 30 PKIs), and basic fibroblast growth factor and histone deacetylase 6 (each with 21 PKIs). Accordingly, preferred non-PK targets of PKIs included popular pharmaceutical targets such as acetylcholinesterase for therapeutic intervention of diseases of the central nervous system; basic fibroblast growth factor, which is a target considered in a variety of therapeutic areas such as oncology or metabolic diseases; and histone deacetylase and bromodomain protein isoforms, both of which are key epigenetic regulators. However, they also included currently unclassified targets. PKIs with activity against well-established pharmaceutical non-PK targets merit immediate consideration for polypharmacology. In addition, PKIs with additional activity against novel targets might aid in exploring their functions. 

Another important finding reported in Table 2 was that the PKIs with activity against preferred non-PK targets were active against largely varying numbers of PKs. For example, these included PKIs with apparent PK selectivity (such as the 32 PKIs shared with acetylcholinesterase and activity against 45 PKs or 18 PKIs shared with thyroid hormone receptor beta-1 and activity against only two PKs) but also highly promiscuous PKIs (such as 30 PKIs shared with FLJ45252 and activity against 353 PKs or 18 PKIs shared with quinone reductase 2 and activity against 291 PKs). Thus, some non-PK targets interacted with PKIs having high PK promiscuity, whereas others interacted with inhibitors of only a few PKs. These observations also have immediate relevance for potential use of PKIs in polypharmacology. 

### 3.8. Drugs with Protein Kinase Activity and Largest Numbers of Non-Protein Kinase Targets

Next, compounds formally qualifying as PKIs according to the micromolar potency threshold with activity against largest numbers of non-PK targets were identified, as reported in Table 3.

The top 10 PKIs with most non-PK targets in Table 3 contained eight drugs. The four compounds with largest numbers (nine to 19 non-PK targets) included three drugs and were each only annotated with a single PK. These drugs were the promiscuous aripiprazole (with 19 non-PK targets), primarily acting as partial agonist of dopamine D2 and serotonin 5HT1A receptors and used as an anti-psychotic for the treatment acute manic bipolar disorders; celecoxib (13 non-PK targets), a cyclooxygenase 2 inhibitor used as an anti-inflammatory; and paroxetine (nine non-PK targets), a serotonin reuptake inhibitor marketed as an anti-depressant. The other top-ranked drugs and clinical compounds were for the most part original PKIs. For example, these chemical entities included crenolanib (with also nine non-PK targets), an investigational drug for cancer treatment, which was annotated with 44 PKIs that were similar to other PKIs used in oncology, such as sorafenib (with five non-PK and 59 PK targets) or erlotinib (four non-PK and 44 PK targets). All of these drugs with largest numbers of non-PK targets and, in part, large numbers of PK targets act through polypharmacology. However, depending on the therapeutic application (for example, diseases of the central nervous system vs. oncology), the balance between non-PK and PK targets significantly varied. 

Furthermore, the comparison in Table 3 also highlights an important aspect inherent in the analysis, that is, differences between primary and secondary targets among compounds with activity against PK and non-PK targets. According to our analysis criteria, all drugs in Table 3 have at least micromolar PK activity and hence qualify as PKIs. However, different from the drugs with multi-PK activity and PKs as primary targets, the first three drugs discussed above are primarily active against other targets and have weak secondary activity against a PK. Hence, one would not classify these drugs as typical PKIs but as drugs with secondary micromolar PK activity. However, in our analysis, only 40 compounds/drugs were identified that were active with nanomolar potency against a primary non-PK target and with micromolar potency against a PK. 

### 3.9. Potency Level Dependence of Protein Kinase Inhibitor–Target Interactions

Finally, we explored how PKI–target interaction might change over increasing compound potency levels. Therefore, interactions between the 447 PKIs and their PK and non-PK targets were monitored by applying different potency thresholds. The results are reported in Table 4. 

Notably, ATP-site-directed PKIs used for cancer treatment are typically required to have low nanomolar potency [1,2], but this requirement does not necessarily extend to polypharmacological use of PKIs in other therapeutic areas, especially involving non-PK targets. At the minimally required potency of 10 µM, 447 active compounds qualified as PKIs with additional targets. These compounds were active against 390 PKs and 210 non-PK targets, as reported above. For increasing potency, the number of PK and non-PK targets generally declined. However, the number of non-PK targets was already reduced from 210 to 134 at 1 µM potency and further declined to 84 and 37 targets at high and low nanomolar potency, respectively. By contrast, at 1 µM potency, only a small reduction in the number of PK targets was observed, and at low nanomolar potency, 146 PKs remained. Thus, PKI interactions with non-PK targets were often of lower potency than with PK targets. Notably, non-PK targets such as cell surface proteins mediating cellular contacts often function on the basis of fast ligand on- and off-rates and transient interactions and thus have typically intrinsically lower affinity for small molecules. Hence, absolute potency values cannot be compared for PKs and non-PK targets, and lower potency for secondary targets might still be relevant for polypharmacology. 

## 4. Conclusions

Over the past three decades, PKIs have become major focal points of drug discovery in different therapeutic areas and a paradigm for the dependence of drug efficacy on polypharmacology. Given that MT activity is a prerequisite for polypharmacology, PKIs binding to the largely conserved ATP site have received much attention because these compounds were generally expected to have multi-PK activity. However, kinome profiling assays and chemoproteomics in cells have shown that many ATP-site-directed PKIs have PK selectivity or even specificity. Other studies have provided evidence that PKIs also interact with non-PK targets. Such insights are relevant for polypharmacology, especially taking into consideration that PKI promiscuity is not only desirable for polypharmacology but also responsible for adverse effects of drugs. While potential activity of PKIs against non-PK targets is of interest from more than one point of view, a systematic assessment of secondary targets of PKIs has not been reported thus far. In this work, we carried out a large-scale, activity data-driven analysis of PK and non-PK targets of PKIs. Care was taken to focus the analysis on high-confidence data to ensure reliability of target assessment. Starting from a large pool of PKIs, a total of 447 PKIs with activity against PK and non-PK targets were identified. These PKIs were active across the human kinome and against a variety of non-PK targets from different classes. PKI–target interactions were systematically analyzed, highly variable activity profiles were detected for these PKIs, and preferred targets were identified. Overall, PKIs with activity against non-PK targets displayed a much wider target distribution than anticipated. In light of these findings, PKIs with activity against PK/non-PK target combinations of interest can be selected for follow-up investigations. Therefore, as a part of our study, the 447 compounds with activity against PKs and non-PK targets and the associated target information have been made freely available. 

## Figures and Tables

**Table 1:** Top 10 protein kinase targets with largest numbers of inhibitors shared with other targets.

ChEMBL Target ID	Name	Group	PKIs	Non-PK Targets
279	Vascular endothelial growth factor receptor 2	TK	53	31
203	Epidermal growth factor receptor erbB1	TK	51	55
1913	Platelet-derived growth factor receptor beta	TK	46	35
5014	Serine/threonine-protein kinase RIPK2	TKL	45	46
4630	Serine/threonine-protein kinase Chk1	GAMK	42	11
1862	Tyrosine-protein kinase ABL	TK	41	32
2973	Rho-associated protein kinase 2	AGC	40	21
2185	Serine/threonine-protein kinase Aurora-B	AUR	38	35
2041	Tyrosine-protein kinase receptor RET	TK	36	36
4439	TGF-beta receptor type I	TKL	35	17

**Table 2:** Top 10 non-protein kinase targets with largest numbers of protein kinase inhibitors.

ChEMBL Target ID	Name	Class	PKIs	PK Targets
220	Acetylcholinesterase	Enzyme	32	45
4105933	Uncharacterized protein FLJ45252	Unclassified	30	353
3107	Basic fibroblast growth factor	Secreted protein	21	26
5465	Histone deacetylase 6	Epigenetic regulator	21	3
3959	Quinone reductase 2	Enzyme	18	291
1947	Thyroid hormone receptor beta-1	Transcription factor	18	2
3879831	Ferrochelatase, mitochondrial	Enzyme	18	296
1741176	X-box-binding protein 1	Unclassified	17	7
1795185	Bromodomain testis-specific protein	Epigenetic regulator	15	179
5378	P_selectin	Adhesion	15	33

**Table 3:** Top 10 protein kinase inhibitors with largest numbers of other targets.

Compound Name	ChEMBL Compound ID	Non-PK Targets	PKs
Aripiprazole	1112	19	1
--	461571	16	1
Celecoxib	118	13	1
Paroxetine	490	9	1
Crenolanib	2105728	9	44
Alisertib	483158	7	11
Flavone	275638	6	1
AZD-5438	488436	5	17
Sorafenib	1336	5	59
Erlotinib	553	4	44

**Table 4:** Protein kinase inhibitor–target interactions at different potency levels.

	pPot ≥ 5	pPot ≥ 6	pPot ≥ 7	pPot ≥ 8
Unique PK targets	390	366	275	146
Unique non-PK targets	210	134	84	37

## Data Availability

Compounds and activity data were obtained from the publicly available ChEMBL database (https://www.ebi.ac.uk/chembl/ (accessed on 1 December 2023)) and BindingDB (https://www.bindingdb.org/ (accessed on 1 December 2023)). The 447 PKIs are made freely available as SMILES representations together with their PK_PD and Non-PK_PD values and target IDs via the following link: https://uni-bonn.sciebo.de/s/RZn52rIjwbPgrR9 (accessed on 13 February 2024).
